# Complete Genome Sequence of the Facultative Methylotroph *Methylobacterium extorquens* TK 0001 Isolated from Soil in Poland

**DOI:** 10.1128/genomeA.00018-18

**Published:** 2018-02-22

**Authors:** Sophia Belkhelfa, Karine Labadie, Corinne Cruaud, Jean-Marc Aury, David Roche, Madeleine Bouzon, Marcel Salanoubat, Volker Döring

**Affiliations:** a Génomique Métabolique, Genoscope, Institut François Jacob, CEA, CNRS, Université d’Évry, Université Paris-Saclay, Evry, France; b Genoscope, Institut François Jacob, CEA, Evry, France

## Abstract

*Methylobacterium extorquens* TK 0001 (DSM 1337, ATCC 43645) is an aerobic pink-pigmented facultative methylotrophic alphaproteobacterium isolated from soil in Poland. Here, we report the whole-genome sequence and annotation of this organism, which consists of a single 5.71-Mb chromosome.

## GENOME ANNOUNCEMENT

The genus *Methylobacterium* contains rod-shaped Gram-negative bacteria and belongs to the family *Rhizobiales*. The members of this group of microorganisms are able to grow on reduced C_1_ compounds such as methane, methanol, and methylamine as sole carbon and energy sources (1, 2). They have been isolated from diverse soil, air, and water environments (3). *Methylobacterium extorquens* TK 0001 was isolated from soil in Poland (4, 5).

*M. extorquens* TK 0001 was obtained from the DSMZ (Braunschweig, Germany) and was grown at 30°C in a minimal medium (DSMZ medium no. 1629) supplemented with 1% methanol. Genomic DNA was purified using the GenElute bacterial genomic DNA kit (Sigma). Genome sequencing was performed at Genoscope, using both Illumina and Nanopore technologies (6, 7). First, the long reads generated by the MinION device (Oxford Nanopore Technologies) were corrected using Canu software (8). The corrected reads were assembled using smartdenovo (https://github.com/ruanjue/smartdenovo), and the resulting assembly was then polished with the help of 2 × 150-bp paired-end reads generated by a MiSeq sequencer (Illumina) with a genome coverage of ~134×. The final assembly was composed of a single contig of 5.71 Mb with a GC content of 68.2%. Automatic functional annotation and comparative genome analysis were performed using the MicroScope platform (http://www.genoscope.cns.fr/agc/microscope) (9). In total, 6,251 genomic objects were identified comprising 6,160 coding sequences (CDSs), 17 miscellaneous RNAs, 59 tRNAs, and 15 rRNAs (5 5S, 5 16S, and 5 23S). The *M. extorquens* TK 0001 genome contains the *mxa* gene clusters (*mxaABCD* and *mxaFJGIR*) coding for methanol dehydrogenase and for enzymes involved in the synthesis of the periplasmic pyrroloquinoline quinone electron carrier. No methylamine dehydrogenase was found. The enzymes for the *N*-methylglutamate pathway, *mgsABC* and *gmaS*, are present, accounting for the growth on methylamine (10). Genes for the serine cycle and the ethylmalonyl-coenzyme A pathway implicated in C_1_ assimilation are specified. *M. extorquens* TK 0001 is a facultative methylotroph growing also on complex carbon sources. Accordingly, the Entner-Doudoroff pathway and the pentose phosphate pathway are predicted, as well as the oxidative tricarboxylic acid cycle.

A comparative genome analysis was performed between TK 0001 and *M. extorquens* strains AM1, PA1, CM4, and DM4 showing that these strains are strongly related. A fraction of 24.3% of the CDSs is specific to *M. extorquens* strain TK 0001, and 61.9% of the CDSs correspond to the core genome of this group of organisms.

### Accession number(s).

This whole-genome project has been deposited in DDBJ/EMBL/GenBank under the accession no. LT962688.
